# Statistical learning under incidental versus intentional conditions

**DOI:** 10.3389/fpsyg.2014.00747

**Published:** 2014-07-10

**Authors:** Joanne Arciuli, Janne von Koss Torkildsen, David J. Stevens, Ian C. Simpson

**Affiliations:** ^1^Faculty of Health Sciences, University of Sydney, Sydney, NSWAustralia; ^2^Department of Special Needs Education, Faculty of Educational Sciences, University of Oslo, OsloNorway

**Keywords:** statistical learning, visual statistical learning, sequence learning, incidental, intentional, implicit learning, explicit learning

## Abstract

Statistical learning (SL) studies have shown that participants are able to extract regularities in input they are exposed to without any instruction to do so. This and other findings, such as the fact that participants are often unable to verbalize their acquired knowledge, suggest that SL can occur implicitly or incidentally. Interestingly, several studies using the related paradigms of artificial grammar learning and serial response time tasks have shown that explicit instructions can aid learning under certain conditions. Within the SL literature, however, very few studies have contrasted incidental and intentional learning conditions. The aim of the present study was to investigate the effect of having prior knowledge of the statistical regularities in the input when undertaking a task of visual sequential SL. Specifically, we compared the degree of SL exhibited by participants who were informed (intentional group) versus those who were uninformed (incidental group) about the presence of embedded triplets within a familiarization stream. Somewhat surprisingly, our results revealed that there were no statistically significant differences (and only a small effect size) in the amount of SL exhibited between the intentional versus the incidental groups. We discuss the ways in which this result can be interpreted and suggest that short presentation times for stimuli in the familiarization stream in our study may have limited the opportunity for explicit learning. This suggestion is in line with recent research revealing a statistically significant difference (and a large effect size) between intentional versus incidental groups using a very similar visual sequential SL task, but with longer presentation times. Finally, we outline a number of directions for future research.

## INTRODUCTION

Many of our beliefs and decisions are generated by brain processes that are not available to consciousness (Eagleman, 2011; Kahneman, 2011). Indeed, it would appear that much of our learning proceeds implicitly. A number of studies have demonstrated that the acquisition of many of our most fundamental abilities, such as motor skills, object recognition, and language, rely on adaptations to regularities in the world that proceed without an intention to learn and without the involvement of conscious awareness (Cleeremans et al., 1998; Perruchet and Pacton, 2006). However, it is also clear that many aspects of the learning of complex abilities can be enhanced with explicit instruction. This raises the question of whether we have two different systems of learning, one implicit and one explicit, and if so, how to tease the two apart (French and Cleeremans, 2002). A number of lesion and brain imaging studies have investigated whether the neural systems supporting explicit and implicit learning are dissociable (e.g., Willingham et al., 2002; Meulemans and Van der Linden, 2003; Reber et al., 2003; Rüsseler et al., 2003; Schendan et al., 2003; Aizenstein et al., 2004; Vandenberghe et al., 2006; Ferdinand et al., 2010; Yang and Li, 2012). So far, findings relating to this issue have been contradictory – complicated by the use of different experimental designs in the various studies (for a review focusing on sequence learning, see Gheysen and Fias, 2012). A related question, explored in the current study, is how awareness of the regularities to be learned influences the learning outcome. Does “trying to learn” and “knowing what to look for” in the input improve learning? Is it possible that the conscious search for regularities in the input might actually interfere with learning?

In an early experiment within the domain of artificial grammar learning (AGL), Reber (1976) compared the effects of giving participants explicit versus implicit instructions. In the explicit condition, participants were directed to search for the complex rules which determined letter orderings, while in the implicit condition no mention of rules was made. Results showed that participants given explicit instructions performed more poorly in all aspects of the experiment than did those given implicit instructions: They memorized the exemplars more slowly, were poorer at determining whether letter-strings were well-formed and tended to invent rules that were not representative of the stimuli. This led Reber (1976) to suggest that one could approach the task with either an explicit or implicit learning mode. He hypothesized that the explicit rule-searching strategy may be effective if the stimulus patterns to be discovered are relatively simple and codable. However, when patterns become more complex and the time to encode them is short, explicit instructions may disrupt performance by encouraging participants to engage in futile rule-search procedures which will frequently lead to invention of rules which are not accurate representations of the stimulus structure. It is worth noting that in the study by Reber (1976) participants who received explicit instructions were not provided with any specific information about the types of regularities that they would be exposed to; they were simply informed that the stimulus materials were determined by rules and that figuring out the rules would be to their advantage.

A number of subsequent studies have failed to replicate Reber’s (1976) finding that explicit instructions can have a detrimental effect on AGL. Studies using the same instructional set as Reber (1976) have generally found that there is no difference in learning between explicitly and implicitly instructed participants (e.g., Dulany et al., 1984; Dienes et al., 1991). However, the few studies which have provided participants with more specific information about the types of regularities to be learned, have tended to find an advantage of explicit instructions (e.g., Howard and Ballas, 1980; Reber et al., 1980). In the study by Reber et al. (1980) participants received explicit instructions regarding the actual schematic structure of the artificial grammar and were given a 7 min “course” on how this structure could be used to generate strings of symbols. Moreover, the time at which this instruction was given was manipulated: one group were given instructions at the outset, one group received them in the middle of the training, and for a third group the instructions were delayed until after they had seen all the training items. A key finding in this study was that the earlier in the training phase that the explicit instruction was given, the larger was the facilitative effect of the instruction.

Within the related literature of serial response time (SRT) tasks, several studies have found that explicit instructions have been beneficial when the pattern is simple, but that they have had no effect when the pattern is more complex or subtle (e.g., Frensch and Miner, 1994; Jiménez et al., 1996). Howard and Howard (2001) observed that intentional instructions to search for a pattern in the input disrupted learning for older, but not younger adults. To explain these results, they suggested that the simultaneous demands of automatic implicit tracking of covariation along with the active search for rules exceeded the processing capacity of older adults. Note, that in these SRT studies, the explicit instructions consisted of general information that the stimuli were governed by rules or followed a systematic pattern, and no specific information about the nature of the rule or pattern was provided (i.e., participants were told they should try to discover these rules).

In sum, AGL and SRT studies have yielded somewhat contradictory findings with regard to the effects of explicit instructions. However, most studies appear to agree that explicit instructions work better (1) when the rules or patterns governing the stimuli are simple rather than complex and (2) when specific information about the nature of the rules or patterns are provided rather than general instructions to search for rules or patterns.

AGL and SRT studies are two important research paradigms used to study implicit learning. A third major paradigm is statistical learning (SL), a term which was coined by Saffran et al. (1996) to describe infants’ ability to segment regularities from the speech stream. In the early years of SL research, the assumption that SL is a form of implicit learning was typically based on indirect evidence. For example, it was often emphasized that participants in SL studies succeed in extracting the underlying statistical structure of the input without receiving any instructions to do so, and that they are generally unable to verbalize the knowledge that is manifest in their familiarity judgments or reaction times (see recent reviews: Romberg and Saffran, 2010; Arciuli and Torkildsen, 2012; Aslin and Newport, 2012). While such findings indicate that SL likely operates implicitly, they are not direct tests of the role of conscious awareness in SL.

Recently, a number of SL studies have taken up the challenge to provide more direct tests of the role of conscious awareness in SL (Kim et al., 2009; Turk-Browne et al., 2009; Franco et al., 2011; Bertels et al., 2012). These studies have adopted a variety of methods from the related literatures on motor sequence learning, AGL, and category learning to assess the conscious status of the acquired knowledge. In an fMRI study of visual SL, Turk-Browne et al. (2009) observed neural responses to statistical structure in areas generally associated with implicit learning, such as the striatum and the medial temporal lobe. Moreover, in a verbal debriefing session, most participants reported no awareness of the statistical structure, and there was no obvious relationship between these verbal reports and accuracy on a behavioral familiarity test. Taken together, these findings were interpreted as evidence that the acquired knowledge was largely implicit. These results are in line with the study by Kim et al. (2009) where subjects’ reaction times showed evidence of implicit learning of visual sequences, but where there was no evidence of learning in a task measuring explicit knowledge. However, Bertels et al. (2012) and Franco et al. (2011) questioned whether the lack of explicit learning in the study by Kim et al. (2009) could be driven by task difficulty, rather than the absence of explicit knowledge. To test this assumption, Bertels et al. (2012) performed a replication of the Kim et al. (2009) study using a simpler task measuring explicit knowledge and found evidence of explicit learning. Moreover, performance in the explicit learning task was associated with subjects’ confidence ratings, suggesting that at least part of their knowledge was available to conscious awareness. Franco et al. (2011) employed an adaptation of the Process-Dissociation Procedure (PDP; Jacoby, 1991) in order to assess whether subjects could consciously manipulate the acquired knowledge in a word segmentation task. The PDP involves contrasting performance in two versions of the same task: an inclusion task where conscious attention and unconscious influences act in concert and an exclusion task where conscious attention and unconscious influences act against each other. Results showed that participants who were able to learn the two artificial languages used in the study, were able to intentionally exclude items from one of two learned languages, suggesting that the acquired knowledge was available to conscious awareness.

The above studies have provided valuable, though somewhat conflicting, evidence about the degree to which knowledge acquired in SL tasks is available to conscious awareness. The studies by Franco et al. (2011) and Bertels et al. (2012) suggest that participants can gain some awareness of statistical regularities in SL tasks, even when no instructions are provided. This finding calls for a distinction between incidental learning conditions (not receiving any instructions) and implicit learning. In other words, participants may not be completely reliant on instructions in order to engage in explicit learning. However, based on the findings in the AGL and SRT studies reviewed above, it would appear that instructions can still have a facilitative effect compared to no instructions, especially if experimenters provide specific information about the regularities to be learned. However, within the research tradition of SL, the effect of explicit instructions on the amount of learning is largely unexplored.

In fact, we know of only three SL studies which have investigated the effects of modifying the instructions given to participants (Kachergis et al., 2010; Hamrick and Rebuschat, 2012; Stevens et al., in press). Two of these studies have employed the cross-situational word learning paradigm, which is often assumed to be a type of SL task (Smith and Yu, 2008). In this paradigm, participants are to infer word-picture mappings when presented with one word and multiple possible referents in each trial, a task which can only be solved if relations between referents and words are tracked across multiple trials.

Kachergis et al. (2010) investigated the effect of different instructions on cross-situational word learning in adults. Instructions were used as a within-subjects variable, so that the same subjects received increasingly explicit instructions during three phases of the experiment: In the first two experimental blocks, participants were simply told to remember each word and each object for a subsequent memory test. In the third block, they were explicitly asked to remember how many times each word and object appeared together during training. In the fourth and final block, they were asked to learn the meanings of the words, a type of instruction which matches those given in previous cross-situational word learning studies. Results showed significant learning even in experimental blocks where the target of learning (word-picture associations) was not mentioned, suggesting that cross-situational learning can proceed incidentally. However, learning was superior when subjects were told explicitly to look for word–picture co-occurrences or to learn word meanings, indicating that strategic inference also plays a role.

Another investigation of cross-situational word learning under incidental and intentional conditions was carried out by Hamrick and Rebuschat (2012). This study differed from Kachergis et al. (2010) by including subjective measures of awareness at the end of the experiment and in using instructions as a between-subjects variable. The intentional group in this experiment received explicit instructions to learn word meanings, and was told that they would be tested afterward. The incidental group was not informed about the purpose of the experiment or that they would be tested afterward. Instead, participants in the incidental group were asked to perform a task that was unrelated to the statistical structure (indicate how many objects in each trial were animate). Participants in both the intentional and incidental groups displayed significant learning, but the learning effect was larger under intentional than incidental learning conditions. However, as the two groups differed both in the instructions they had received before the training phase and in the cover task that was only given to the incidental group, the differences in learning effects could be due to either of these manipulations.

The above studies have provided evidence that performance in a cross-situational learning task is aided by explicit instructions. There is, however, some dispute as to whether participants use SL mechanisms in this task, or whether they employ hypothesis testing strategies to learn the word-referent mappings (e.g., Medina et al., 2011; Trueswell et al., 2013). Thus, it is worthwhile to conduct studies with other types of SL tasks before drawing conclusions about the effect of explicit instructions on SL.

A study conducted by Stevens et al. (in press), in the same lab as the current study, was designed to assess the impact of concurrent physical movement on learning when SL is performed under incidental versus intentional conditions. The physical movement task required participants to cycle on a stationary bike during the familiarization phase – participants in one group cycled at moderate intensity while another group of participants engaged in resistance free cycling. In addition there were control groups where participants were not engaged in concurrent physical movement but still sat on a stationary bike during the familiarization phase. All groups engaged in the test phase while seated at a desk. The aims of that study were quite different to the aims of the current study. However, as in the current study, the Stevens et al. (in press) study employed a visual sequence learning task, the embedded triplet paradigm, and used the same kinds of stimuli reported here, cartoon-like figures loosely described as aliens. While some of the key findings of the Stevens et al. (in press) study are not relevant to the discussion presented here the findings relating to the control groups are relevant. Stevens et al. (in press) included two control groups: a control group in Experiment 1 that received no instructions to learn, and a control group in Experiment 2 that received explicit guidance on learning the embedded triplets during familiarization. Using a stimulus presentation time of 800 ms, the Stevens et al. (in press) study revealed that both control groups showed significant learning (above 50%), but that the intentional group demonstrated higher learning than the incidental group.

The aim of the present study was to further investigate how explicit versus implicit test instructions affect performance in a typical SL task. As the three previous studies mentioned above (Kachergis et al., 2010; Hamrick and Rebuschat, 2012; Stevens et al., in press), we studied the effect of having conscious knowledge of the statistical structure to be learned prior to learning. However, unlike two of the previous studies which investigated the ability to learn label-object pairings, but like the study by Stevens et al. (in press), the present study examined sensitivity to sequential relationships. We employed a visual paradigm which has been used extensively in the SL literature: the embedded triplet paradigm (e.g., Fiser and Aslin, 2002; Turk-Browne et al., 2005; Brady and Oliva, 2008; Turk-Browne et al., 2009; Arciuli and Simpson, 2011, 2012a,b). Using a between-subjects design we compared the detection of embedded triplets in an incidental version of the task (i.e., implicit as there was no instruction to learn) versus an intentional version of the task (i.e., explicit as there was clear instruction regarding what to learn). The instructions given in the present study had the two characteristics that have been found to facilitate learning in AGL studies and SRT tasks (see above): (1) the regularities in the input were simple rather than complex (embedded triplet sequences) and (2) participants were provided with specific information about the nature of the regularities rather than general instructions to look for patterns.

Importantly, the only difference between the intentional and incidental versions of the experiment was the instructions given before the familiarization phase. Thus, any difference in performance between the incidental and intentional groups could be attributed to the instructions alone. Another important feature of the present experiment is the presentation speed. In order to provide maximum contrast with the long presentation time (800 ms) used by Stevens et al. (in press), we used a stimulus presentation time of 200 ms. Two previous studies have shown that SL can occur at this very fast stimulus presentation rate of 200 ms (Turk-Browne et al., 2005; Arciuli and Simpson, 2011). However, the effect of instructions on SL has not been investigated at fast stimulus presentation rates.

## MATERIALS AND METHODS

### PARTICIPANTS

Approval to conduct the study was granted from the University of Sydney Human Research Ethics Committee. A total of 23 female undergraduates from the University of Sydney ranging in age from 18–25 years volunteered to take part in the current study in return for course credit. Participants were randomly allocated to either the incidental-or intentional-instruction condition.

### MATERIALS AND PROCEDURE

The design of the embedded triplet SL task was similar to that reported in three previous studies by Arciuli and Simpson (2011, 2012a,b). It was comprised of two separate phases: a familiarization phase followed by a test phase. Stimuli were eighteen cartoon-like figures, loosely described as aliens, sourced from the website http://www.clipartconnection.com/en/^1^. As in previous published studies by Arciuli and Simpson (2011, 2012a,b) and the study by Stevens et al. (in press) these cartoon-like figures were deliberately chosen because they are not easy to verbalize. That is the cartoon-like figures are not easily characterized based on their defining features. This can be confirmed by examining the stimuli that are supplied in the Appendices of the previously published work. Twelve of the aliens were chosen as stimuli for the experiment, and these were divided into four groups of three (four base triplets) hereafter referred to as ABC, DEF, GHI, and JKL. The remaining six aliens were used only during instructional and practice phases as examples. This ensured that viewing specific stimuli during the instruction phase did not influence the degree of learning during the familiarization phase. The 12 aliens used for testing, along with the triplet groupings are shown in Appendix A.

#### Familiarization phase

The familiarization phase consisted of a continuous stream of stimuli, with each alien shown in isolation in the center of the display against a white background. Each alien was visible for 200 ms with an inter-stimulus-interval (ISI) of 200 ms. Returning to the point about our alien stimuli being chosen because they were not easily verbalizable we would like to emphasize that a stimulus presentation time of 200 ms using unfamiliar complex figures – in our case, cartoon-like aliens – leaves very little opportunity to attempt to name (i.e., verbalize) each figure. Each base triplet was selected for inclusion 24 times each (giving a total of 96 triplets). For six of these 24 instances, one of the aliens was presented with a yellow background, instead of the usual white background, in order to provide a cover task. The cover task ensured that participants paid attention to the familiarization stream as they were required to press a button whenever they saw an alien with a yellow background (described in the instructions as a “radioactive” alien). Radioactive aliens were counterbalanced among the three aliens within each triplet so that each appeared radioactive on two occasions. In total, the familiarization stream consisted of 288 individual aliens, with each of the 12 aliens appearing 24 times each, and 24 of the aliens appearing as radioactive.

The order of the triplets within the familiarization stream was randomized with the single restriction that the same base triplet could not appear consecutively (e.g., ABCABC). Due to these constraints, four different pre-randomized familiarization lists were created with each participant viewing one of these four lists. After three trials in which participants practiced identifying the radioactive aliens, participants in the explicit instruction group were given the additional information that the aliens usually appeared in groups of three, and that they would be asked later if they could identify these groupings. Participants in the implicit learning condition were naïve as to the existence of these groupings.

#### Test phase

For the test phase four new triplets were created, with each containing one alien from each of three base triplets. Due to the ordering constraints, these new triplets never appeared in the familiarization stream. These four impossible triplets are referred to as AEI, DHL, GKC, and JBF. For each test trial, one actual base triplet was displayed along with one impossible triplet. The aliens in each triplet were presented one at a time using the same presentation time and ISI used in the familiarization phase with a 1,000 ms gap separating the base triplet from the impossible triplet. After all six aliens had been presented a new screen appeared which prompted participants to identify which of the two triplets had appeared previously in the familiarization phase, with no time constraints imposed. Each base triplet was presented with each impossible triplet on four separate occasions with the presentation order counterbalanced. Across the 64 test trials each base triplet was seen 16 times and each impossible triplet was seen 16 times. This insured that if any SL took place during the test phase itself, the opportunities to learn were equal for both types of triplet. Each participant received a different random order for the test trials. Item presentation and data collection in both phases were controlled using E-prime software (version 2; Schneider et al., 2002).

## RESULTS

In analyzing our results, data from the familiarization phase were inspected to determine the number of “radioactive” aliens successfully identified. Correct identification was defined as pressing a button within 3 s of the onset of these aliens. Participants who failed to identify at least 18 of the 24 “radioactive” aliens were excluded on the grounds that they may not have been attending to the familiarization stream. As a result of this screening, one participant was excluded from the explicit instruction condition.

Data from the test phase were inspected to determine the number of base triplets successfully identified by each participant (as a percentage of the overall number of trials in the test phase). Within each of the two instruction conditions, participants who scored ±2 SDs from the mean of that condition were excluded. This resulted in the exclusion of one participant from the implicit instruction condition who had an excessively low score. Table**Table 1** summarizes the test performance for the retained participants.

**Table 1:** Mean percentage of triplets successfully identified in the test phase for retained participants in each instruction condition.

Learning	*n*	Test phase			
Condition		Mean	*SD*	Min	Max
Implicit	11	58.5	11.8	40.6	75.0
Explicit	10	63.9	17.3	40.6	89.1
					
Total	21	61.1	14.6	40.6	89.1

Chance performance is reflected in correct identification of 50% of the base triplets presented during the test phase. SL can be said to have occurred if significantly more than 50% of base triplets were correctly identified. Overall, the 21 participants demonstrated significant SL (mean 61.1%, one-sample *t*-test *t*[20] = 3.49, *p* = 0.002, *d* = 0.76). To determine if SL occurred within each of the instruction conditions two separate one-sample *t*-tests were performed comparing the SL elicited in each instruction condition with the chance rate of 50%. Both tests proved to be significant; *t*(9)_Explicit_ = 2.54, *p* = 0.032, *d* = 0.80; *t*(10)_Implicit_ = 2.41, *p* = 0.037, *d* = 0.73. To determine if instruction condition affected the amount of SL, an independent samples *t*-test was conducted. This was not significant, *t*(19) < 1, *p* = 0.411, suggesting that the amount of SL was similar for each learning condition. The effect size was small (*d* = 0.36).

To strengthen the argument that there was no meaningful difference between the two groups we also performed Bayesian analysis incorporating Markov chain Monte Carlo estimation (Kruschke, 2013). This technique provides distributions of likely means, standard deviations, and effect sizes, given the data. From these distributions, a range of credible values can be estimated. Specifically, we used these distributions to test group differences against a difference of 0. From this analysis the mean difference between the two groups was estimated to be 5.22 with a 95% highest density interval (HDI) of (-10.6, 21.3). Had a robust difference existed between the two groups, zero would have fallen outside of the HDI. Thus, the Bayesian analysis indicates that there is no credible difference between the means, which is in accordance with the *t*-test result.

## DISCUSSION

Our results revealed that there was no reliable difference in visual sequence learning in the incidental versus intentional versions of the task. This result stands in opposition to findings in previous studies which have compared SL under incidental and intentional conditions (Kachergis et al., 2010; Hamrick and Rebuschat, 2012; Stevens et al., in press). All of these previous studies observed better performance in the intentional than in the incidental version of the task. There is, however, one important difference between the current study and these three previous studies. The current study used a short stimulus presentation time, where each visual stimulus was visible for only 200 ms. In the cross-situational word learning studies by Kachergis et al. (2010) and Hamrick and Rebuschat (2012) the stimuli were visible for 6 and 14 s, respectively. In the study by Stevens et al. (in press) which used the same embedded triplet paradigm and the same cartoon-like aliens that we used in the current study, each stimulus was visible for 800 ms. This means that, of the three previous studies mentioned above, even in the study with the shortest presentation times (i.e., Stevens et al., in press), participants were given four times longer to process each image than the participants in the current study. Thus, it seems possible that the short stimulus presentation time used in the current study precluded the use of explicit strategies to learn the embedded patterns in the familiarization stream.

The findings in the present study are in line with a number of studies in the fields of AGL and SRT tasks which have found no difference in the amount of learning between explicitly and implicitly instructed subjects (e.g., Dulany et al., 1984; Dienes et al., 1991; Jiménez et al., 1996). However, the instructions given in those previous studies were generally much less specific than in the present study. The few studies within these paradigms that have provided subjects with specific information about the rules or patterns to be learned, have found better learning in the explicit than the implicit condition (Howard and Ballas, 1980; Reber et al., 1980). In terms of the specificity of instructions, the latter studies are more directly comparable to the present study. It should be noted, though, that in the Reber et al. (1980) study, explicitly instructed participants were given a 10 min training procedure – not only were they informed about the specific rules, they were also asked to generate strings based on the rules. Moreover, the stimulus presentation times were much longer than in the present study. The experiment by Howard and Ballas (1980) used presentation times more comparable to the present study (320 msec for each stimulus item), but in that experiment auditory stimuli were used, and as will be suggested below, presentation time might have different effects for visual versus auditory stimuli.

One way of examining the hypothesis that short presentation times may hinder the use of explicit strategies, is to further investigate SL under incidental and intentional conditions using a variety of stimulus presentation times. We are not aware of any studies in the field of SL, or in the related fields of AGL, probability learning, motor sequence learning, or category learning that have investigated a possible interaction between instructions and presentation speed. Some results indicate, however, that it may be more fruitful to investigate this question in the visual rather than auditory domain, as visual SL performance (at least for sequential stimuli) appears to be more affected by presentation speed. A study by Conway and Christiansen (2009) comparing SL in adults in different presentation formats (visual input distributed spatially, visual input distributed sequentially, and auditory input distributed sequentially) and at different presentation speeds, found that faster presentation rates (going from four elements per second to eight elements per second) led to a decline in SL only for the visual input that was presented sequentially. Similar findings were reported in a study by Arciuli and Simpson (2011) which used the embedded triplet paradigm to study visual sequence learning in 5–12 year old children – learning increased with slower presentation times for stimuli.

An alternative interpretation of the findings in the current study is that participants in the intentional group were in fact using explicit learning strategies based on the instructions, and that participants in the incidental group were able to gain enough awareness of the regularities in the input to develop comparable explicit strategies. We did not ask participants in the incidental group whether they had gained awareness of any embedded patterns or whether they had tried to use strategies, so we cannot refute or confirm this possibility. In a visual embedded triplet task where no explicit instructions were given, Bertels et al. (2012) found that subjects gained some conscious knowledge of the regularities in the input, even though the presentation rate was as fast as in the current study (200 ms). More specifically, data from a confidence judgment task was compared to actual performance, and showed that participants judged that they remembered the correct sequence 54% of the time when they were actually correct, and 43% of the time when they were incorrect, a difference that was statistically significant. Based on this result, it is possible that the participants in the incidental condition of the present study gained some conscious awareness of the stimuli. However, it is unlikely that incidentally instructed participants gained awareness of the regularities to the degree necessary to employ learning strategies comparable to the intentionally instructed group. Results from previous AGL studies suggest that intentional instructions of sufficient specificity aid the use of explicit learning strategies and lead to better performance (Howard and Ballas, 1980; Reber et al., 1980). As discussed in more detail below, results from the SL study by Stevens et al. (in press) are in line with these findings.

Stevens et al. (in press) explored the effects of concurrent physical movement on SL. However, that study included two control groups that are useful for the purposes of cross-study comparison. Stevens et al. (in press) included a control group in Experiment 1 that received no instructions to learn (*n* = 12), and a control group in Experiment 2 that received explicit guidance on what to learn (*n* = 10). These control groups are comparable with the incidental and intentional conditions reported in the current study. Each of the control groups in the study by Stevens et al. (in press) showed statistically significant learning (above 50%). However, the intentional group demonstrated higher learning. A direct test of whether this difference was statistically significant was not reported in the Stevens et al. (in press) paper. However, we have re-analyzed that data and are able to report that an independent samples t-test revealed a significant difference, *t*(20) = 22.06, *p* < 0.001. It is important to note that the effect size associated with the difference was large (*d* = 2.05). Participant numbers in the Stevens et al. (in press) paper (*n* = 22 in total) are almost identical to the participant numbers in the current study (*n* = 21 in total). Yet, there is a significant difference between means accompanied by a large effect size in the Stevens et al. (in press) data set while, in the current study, we observed a non-significant difference accompanied by a small effect size.

In sum, both AGL studies and a study using a comparable SL paradigm have shown that specific explicit instructions can improve learning outcome. Thus, if the intentional group in the present study had been able to benefit from the instructions, it seems unlikely that the incidental group would have managed to perform comparably to the intentional group. Consequently, we reiterate that one possible interpretation of the discrepancy in results between the Stevens et al. (in press) and the current study is that with a slow stimulus presentation speed (800 ms per stimulus), the participants in the intentional control group in the Stevens et al. (in press) study were able to learn the embedded triplets explicitly. By contrast, with a fast stimulus presentation speed (200 ms per stimulus) the participants in the intentional group in the current study were less able to exploit explicit learning strategies.

Taken together, the findings of SL studies comparing learning under intentional and incidental conditions suggest that explicit learning is superior to implicit learning, at least when the regularities to be learned are relatively simple and stimulus presentation times are not fast. However, we think that there may be other factors that should be taken into consideration. For example, much of the SL research, including that reported here and by Stevens et al. (in press) assesses *immediate* learning. Much less is known about *retention* of the learned material. The handful of studies that have examined rates of forgetting for implicit SL suggest slow decay rates, contrary to what would be expected if the knowledge was explicit (Kim et al., 2009; Arciuli and Simpson, 2012b). It would be interesting to investigate rates of forgetting in incidental versus intentional groups using short versus long presentation times. Similarly, we reiterate that much of the SL research reports on stimuli that are associated with relatively simple statistical regularities. One might speculate that as the complexity of the statistical regularities increases, it becomes difficult, perhaps even impossible, to explicitly communicate these regularities to participants in a coherent way, let alone observe participants’ explicit learning of these regularities. Comparison of incidental versus intentional conditions using SL tasks with simple versus complex statistical regularities is another interesting avenue for future research.

In conclusion, we propose that SL mainly operates implicitly when presentation times are short. Under these conditions, provision of explicit instruction regarding the nature of embedded regularities to be learned does not appear to enhance learning. With longer presentation times differences between SL tasks administered under intentional versus incidental learning conditions may emerge and, under such conditions, explicit learning may be more effective than implicit learning, possibly contingent upon the statistical regularities to be learned being relatively simple.

## Conflict of Interest Statement

The authors declare that the research was conducted in the absence of any commercial or financial relationships that could be construed as a potential conflict of interest.

## Footnotes

1 Access to this website is via subscription. Images can be legally downloaded from this website during the subscription period and can be used in print or electronic form indefinitely.
